# Control of PDGF-induced reactive oxygen species (ROS) generation and signal transduction in human lens epithelial cells

**Published:** 2007-03-14

**Authors:** Kate Chao-Wei Chen, You Zhou, Wei Zhang, Marjorie F. Lou

**Affiliations:** 1 Departments of Biochemistry, University of Nebraska-Lincoln, NE; 2 Veterinary and Biomedical Sciences, University of Nebraska-Lincoln, NE; 3 The Redox Biology Center, University of Nebraska-Lincoln, NE; 4 Beijing Institute of Ophthalmology, Beijing TongRon Hospital and Capital University of Medical Sciences, Beijing, China; 5 Department of Ophthalmology, University of Nebraska Medical Center, Omaha NE

## Abstract

**Purpose:**

The mitogenic action of PDGF has been shown to associate with reactive oxygen species (ROS) generation, but the mechanism leading to ROS production and subsequent cell proliferation is not clear. We investigated the upstream membrane-bound target proteins involved in PDGF-stimulated signal transduction in human lens epithelial cell (HLE B3), using specific inhibitors and transfected cells.

**Methods:**

PDGF (1 ng/ml)-stimulated ROS generation was measured using fluorescent reaction of DCFDA by confocal microscope in live HLE B3 cells. Western blot analysis was used to determine the activated MAP kinases in cell lysates. Specific inhibitors used in this study were: AG1296 for PDGF receptor (PDGFR); AG1517 for EGF receptor (EGFR); pertussis toxin for cytokine-binding G protein coupled receptor (GPCR); PP1 for Src-family kinases; LY294002 for phosphatidylinositol-3 kinase (PI3K). Small GTP-binding proteins Rac and Ras were studied using transfectants of dominant negative Rac (Rac N17), Ras (Ras N17) or constitutively active Rac (Rac V12). Cell proliferation was quantified using BrdU incorporation method.

**Results:**

Inhibitions of PDGF receptor kinase, the docking protein component Src-family kinases, and the survival element PI3K all eradicated PDGF-stimulated ROS production and corroborated with the suppressed cell growth. These inhibitions also attenuated the activated ERK1/2, JNK, and Akt, all downstream targets of the above factors. Interestingly, inhibiting GPCR or EGFR also showed the same effect but to a lesser degree. Co-inhibiting receptors to PDGF and EGF with or without co-inhibiting GPCR eradicated the PDGF signaling system completely. Transiently transfected cells with plasmid from small GTP-binding proteins Rac N17 or Ras N17 diminished PDGF action in ROS generation, cell proliferation and MAP kinase activation, while cells with Rac V12 enhanced the PDGF effect.

**Conclusions:**

Our data clarified the potential mechanism of PDGF signaling in the lens epithelial cells, in which concerted efforts of the upstream components of PDGF receptor kinase, Src-family kinases, PI3K, Rac, and Ras proteins are required. This report also provided novel findings that GPCR and EGF receptors may control PDGF signaling in the lens epithelial cells via integrative signaling and transactivation mechanisms, respectively.

## Introduction

Reactive oxygen species (ROS) are recently recognized to be beneficial to cells in regulating signal transduction in plants and animals, a process called redox signaling [1-6]. This process is initiated by a burst of intracellular ROS generation stimulated by various growth factors and cytokines. ROS in turn participate and regulate diverse downstream signaling pathways leading to specific cellular functions [7-13]. One of the targets for ROS in vivo is the reversible oxidation of phosphatases, which together with protein tyrosine kinases are responsible for maintaining a normal protein tyrosine phosphorylation-dephosphorylation homeostasis in cell signaling in vivo [5,14].

Studies have revealed that the likely source for ROS generation is the membrane-bound superoxide-generating enzyme NADPH oxidase [15]. The activity of NADPH oxidase is controlled by a group of enzymatic components, including the small GTP-binding proteins Rac and Ras. Rac may be involved in regulating the levels of ROS after ligand-evoked activation [16-19] or it may serve as an activator for cytosolic phospholipase A_2_, which releases arachidonic acid from the membrane for cellular functions [20]. Ras is known to act as a switch to regulate signal transduction pathways that control cell proliferation, differentiation, organization of actin cytoskeleton, intracellular transport and survival [21-28]. Ras can be recruited and anchored onto the inner surface of cell membrane where it is modified and activated to Ras-GTP [29,30], as a cellular redox regulator [16,31].

Among the various growth factors, PDGF is well-studied in many cell types and used by many as a model system. PDGF family consists of four gene products that form five dimeric isoforms: αα, ββ, αβ, CC and DD [32]. Each isoform acts via two receptor tyrosine kinases of PDGFRα and PDGFRβ inducing dimerization of receptors and autophosphorylation of distinctive tyrosines in the intracellular domain of the receptor. The specifically phosphorylated tyrosine allows docking and subsequent activation of a series of responding molecules containing Src homology 2 or SH2 domains [33]. These include Src family kinases, phosphatidylinositol-3-kinase (PI3K), phospholipase Cγ (PLCγ) and small GTP-binding protein Ras [34-36]. The specific binding of these molecules can initiate signaling pathways leading to cell proliferation and motility [37,38]. In response to PDGF stimulation, there is a crosstalk between signaling pathways leading to cell growth. For example, Ras and PI3K have been demonstrated to interact with, and mediate, each other [39,40]. Small GTP-binding proteins Rac, Ras, and Rho have also been proven to activate each other in several cell types [41-43].

In addition to growth factor receptors, the cells have G protein coupled receptors (GPCRs) which consist of a group of integral membrane proteins. These receptors respond to diverse external stimuli and subsequently interact with their respective large G proteins to initiate various downstream pathways upon ligand binding [44]. GPCR is proposed to interact with protein tyrosine kinase receptor (PTKR) binding during growth factor stimulation, and subsequently activating Src-family kinases and other upstream signaling components. It has been reported that GPCR can combine with PDGFR to form a functional signaling complex in human embryo kidney cells [45]. However, whether GPCR interacts with PDGF receptor in the lens is not clear.

Many of the PDGF isoforms are potent mitogens for lens growth and development [46], including the recently discovered effective PDGF-D [47]. We have used the PDGFββ isoform in the past and found that human lens epithelial (HLE) cells responded to its mitogenic action, and that action was ROS-dependent. PDGF-stimulated ROS generation and the downstream signal transduction in HLE cells could be abolished when cells were preloaded with antioxidants or free radial scavengers. In addition, a range of low level exogenous hydrogen peroxide could mimic the mitogenic effect of PDGF [48]. The membrane NADPH oxidase is known to be the major source of ROS in other cell types, and this enzyme has been found in both the lens and lens epithelial cells [49,50]. Our preliminary studies have also shown that NADPH oxidase was the main source of such ROS generation [51]. The mechanism of PDGF signaling is largely unknown. To continue our quest for the understanding of PDGFββ signaling in the lens, we focus on identifying the target proteins after PDGF receptor (PDGFR) binding and prior to ROS generation by using the PDGFR-rich HLE B3 cells as a model. The upstream factors included PDGFR, Src-family kinases, PI3K and small GTPase Ras and Rac. We also examined the possible crosstalk between GPCR and PDGFR, and between EGFR and PDGFR in the lens epithelial cells. Using both specific inhibitors, we found that inhibiting either PDGFR, Src-family kinases or PI3K prevented ROS production, MAPK activation or cell proliferation in PDGF-stimulated cells. Dominant negative Ras or Rac transfected HLE B3 cells switched off ROS generation and the downstream signals while constitutively active Rac would enhance them. Our data also suggest the presence of crosstalk between PDGF receptor and the G-protein coupled receptor, and also between PDGF receptor and EGF receptor in the lens epithelial cells.

## Methods

Lipofectamine^TM^ Transfection, Plus Reagent, pcDNA 3.1+ and 2',7'-dichlorodihydrofluorescin diacetate (DCFDA) were purchased from Invitrogen Corporation (Carlsbad, CA). Rac1 N17, Rac1 V12, and H-Ras N17 (HA-tagged) were purchased from Guthrie cDNA Resource Center (Sayre, PA). Platelet-derived growth factor (PDGF) ββ homodimer (human recombinant), Pertussis toxin (*Bordetella pertussis*) and AG1296, 4-[(3-bromophenyl) amino]-6,7-dimethoxyquinazoline (AG1517) were obtained from Calbiochem (San Diego, CA). 2-(4-morpholinyl)-8-phenyl-4H-1-benzopyran-4-one (LY294002) and 4-amino-5-(4-methylphenyl)-7-(t-butyl) pyrazolo [3,4-d] pyrimidine (PP1) were purchased from BIOMOL International, LP (Plymouth Meeting, PA). Cell Proliferation ELISA 5-bromodeoxyuridine or BrdU (chemiluminescent) Kit, and Cytotoxicity Detection Kit (lactic acid dehydrogenase or LDH kit) were purchased from Roche Applied Sciences (Indianapolis, IN). Phospho-ERK1/2 monoclonal antibody, phospho-p38, phospho-SAPK/JNK, and phospho-Akt polyclonal antibodies were all obtained from Cell Signaling Technology Inc. (Beverly, MA). HA antibody and horseradish peroxidase-conjugated secondary antibodies were purchased from Santa Cruz Biotechnology, Inc. (Santa Cruz, CA). Anti-G3PD (Glyceraldehyde-3 phosphate dehydrogenase) antibody was purchased from Research Diagnostics Inc. (Flanders, NJ). SuperSignal West Pico Substrate was purchased from Pierce Biotechnology Inc. (Rockford, IL). All other chemicals and reagents were of analytical grade.

### Cell Culture

Human lens epithelial cell line, HLE B3, was kindly provided by Usha Andley of Washington University (St. Louis, MO). Cells were grown and maintained in medium consisting of MEM supplemented with 20% FBS and 50 μg/ml of gentamicin in a humidified 5% CO_2_ incubator. Medium was changed every four days. For PDGF stimulation, cells were gradually deprived of serum by first incubating in medium with 2% FBS overnight, then replacing into serum-free medium for 30 min prior to each experiment.

### Cell Transfection

Lens epithelial cells are known to contain several small GTP-binding proteins (23-27 kDa) including Rac-1 and H-Ras [52]. In this report, we used Rac-1 and H-Ras throughout the experiments. Rac N17, Rac V12 or Ras N17 constructed with HA-tag (Hemagglutinin epitode) was transfected into HLE B3 cells by using Lipofectamine^TM^ Transfection with Plus Reagents. Cells were seeded and cultured overnight in 20% FBS-containing MEM. Lipofectamine transfection with Plus Reagent, and plasmid DNA were added onto cells in serum-free medium for 3 h. The cells were cultured in 20% FBS-containing medium for 48 h and then geneticin (800 μg/ml) was used to select the transfected cells. After selection, transiently transfected cells were maintained in medium containing 400 μg/ml geneticin.

### Quantitative image analysis of intracellular ROS in live cells by confocal microscopy

Human lens epithelial cells (wild-type or transfected) were gradually deprived of serum as described above. Cells were then loaded with 50 μM of DCFDA for 5 min in the dark in a CO_2_ incubator before stimulating by PDGF, following the method described in Chen et al. [48]. In brief, after loading DCFDA, the dye was removed and cells were washed twice with MEM-HEPES before image collection. Un-stimulated cells exposed to UV light (from confocal microscope) were used as the positive control for ROS reaction and dye-loading quality, while cells without growth factor were used as controls.

All confocal imaging analyses were performed with a BioRad MRC1024ES confocal laser scanning microscope. The real time imaging on cells preloaded with DCFDA was carried out with a 40X water immersion lens using the 488 nm excitation laser line and simultaneous dual display mode (522 nm emission and phase-contrast) of the BioRad LaserSharp imaging program. Each image series was collected under the same system settings, such as the black level and the magnification, using 10% laser intensity and 0.5 s/frame (512x512) scan speed. Five random images were collected as determined under phase-contrast/transmitting channel for focus and confluence of cells within the frame (usually covering >90% areas of 512x512 image frame that yields about 90-100 cell per frame) at each of the given time points. Average fluorescence intensities were determined from each of the images using the BioRad LaserSharp program. Data from five random images within a given time point were pooled and averaged again to obtain the mean fluorescence intensities.

### Treatment of cells by inhibitors

Cells were gradually deprived of serum as described above and then treated with various inhibitors before subjecting to PDGF stimulation. For the treatment of PDGF receptor inhibitor 6,7-dimethoxy-3-phenylquinoxaline (AG1296), EGF receptor inhibitor 4-[(3-Bromophenyl)amino]-6,7-dimethoxyquinazoline (AG1517), G protein coupled receptor (GPCR) inhibitor pertussis toxin (Ptx), Src family kinases inhibitor, 4-amino-5-(4-methylphenyl)-7-(t-butyl) pyrazolo [3,4-d] pyrimidine (PP1), or phosphatidylinositol 3 kinase (PI3K) inhibitor, 2-(4-morpholinyl)-8-phenyl-4H-1-benzopyran-4-one (LY294002), serum-starved cells were loaded with AG1296 (20 μM, 30 min or 2.5 h), AG1517 (1 μM, 2.5 h), Ptx (250 or 500 ng/ml, 1 or 2.5 h), PP1 (10 μM or 20 μM, 30 min), or LY294002 (15 μM, 30 μM, 40 μM, 30 min), respectively either individually or in combination prior to PDGF addition.

We chose these inhibitors as it has been shown previously in other cell types, the beta receptor of PDGF, the Src-family kinases, PI3K and GPCR are all initial downstream targets for PDGF-binding at the receptor [45,53-58]. AG1296 is a potent and specific inhibitor for PDGF receptor tyrosine kinase [59]. This quinoxalin-type tyrphostin acts as an ATP-competitive inhibitor of the receptor kinase, but does not interfere with ligand binding or receptor dimerization. However, it does affect the autophosphorylation of PDGF receptor [60]. AG1517 is a specific inhibitor for EGF receptor that acts by competing for ATP binding sites with EGF. AG1517 can rapidly suppress autophosphorylation of EGF receptor and therefore selectively impedes EGF-mediated cellular processes. PP1 is a potent Src-family-selective tyrosine kinase inhibitor [61], whose inhibitory activity appeares to act through ATP-competitive binding by interacting with the active site of Src tyrosine kinases [62]. LY294002 is a flavonoid quercetin based compound, which can completely abolish PI3K activity by targeting the ATP-binding site of the catalytic unit p110 only [63,64], and has no inhibitory effect on other ATP-requiring tyrosine kinases [64]. We also chose to inhibit GPCR as recent reports have indicated that GPCR plays a major role in growth factor signaling [45,57,65-67]. Ptx, which is a secretory product of *Bordetella pertussis*, prevents guanyl nucleotide-induced dissociation of Giα from Giβγ subunits, a process that is crucial for the function of GPCR [68].

### Cell proliferation detected by 5-bromodeoxyuridine (BrdU) incorporation assay

5-Bromodeoxyuridine (BrdU), a chemical analog of thymidine, was used in BrdU incorporation assay for cell proliferation. The method of using monoclonal antibodies directed against BrdU is often used to measure DNA synthesis, in which the amount of BrdU incorporated into cultured cells can be quantified chemiluminescently in a luminometer.

BrdU incorporation assay was performed according to the manufacturer instruction. Briefly, cells were seeded onto black 96-well plates overnight and medium was replaced with 2% FBS-containing MEM for another overnight. BrdU labeling solution (final concentration of 10 μM) and PDGF (1 ng/ml) were added onto serum-starved cells and incubated for 1 h. After labeling, cells were fixed and incubated with anti-BrdU-POD. Excess antibody was removed by washing the cells with 1X PBS, and the chemiluminescence was determined by luminometer equipped with automatic substrate injectors (FLUOstar OPTIMA manufactured by BMG Labtech). The specific chemiluminescence was expressed as RLU/second.

### Western blot analysis

The serum-deprived cells (wild-type or transfected) preloaded with or without various inhibitors, as described above, were treated with PDGF at various time periods. Western blot analysis was performed as described in Chen et al. [48].

### Protein determination and statistical analysis

Protein concentration was determined by BCA microanalysis [69]. Statistical analysis was done using Student's t-test. An associated probability (p) value of <0.05 was considered significant.

## Results

### Effect of inhibition on PDGF receptors, Src-family kinases, PI3K, and GPCR on PDGF-stimulated ROS generation in human lens epithelial (HLE) B3 cells

**Figure 1:**
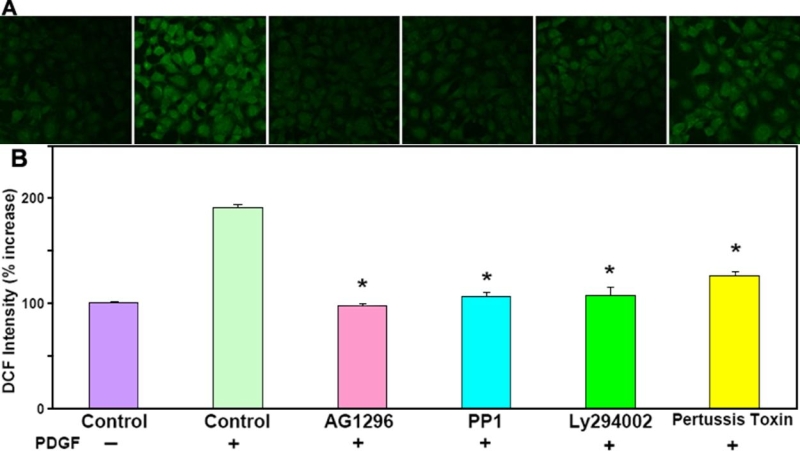
Confocal images of intracellular ROS generation upon platelet-derived growth factor (PDGF) stimulation in live HLE B3 cells. Live HLE B3 cells were preloaded with DCFDA (50 μM) to capture the ROS generated upon PDGF (1 ng/ml) stimulation. Confocal cell images represent a random field after PDGF exposure. For further details see Methods. **A**: Panels from left to right in following conditions: control cells (no PDGF); control cells (with PDGF); PDGF-stimulated cells pretreated with PDGF receptor inhibitor, AG1296 (20 μM, 30 min); PDGF-stimulated cells pretreated with Src inhibitor, PP1 (10 μM, 30 min); PDGF-stimulated cells pretreated with PI3K inhibitor, LY294002 (15 μM, 30 min); and PDGF-stimulated cells pretreated with inhibitor to G-protein coupled receptor, pertussis toxin or Ptx (500 ng/ml, 1 h). **B**: Bar graph corresponding to the confocal images in **A**. The DCF fluorescent intensity was quantified using Confocal Assistant 4.02, and expressed as percent increase normalized to control (100%). The data are expressed as mean±SD with n=3. Each p value was obtained by using inhibitor against control (with PDGF). The asterisk indicates a p<8x10^-5^.

To examine the effect of PDGF binding on its downstream targets with and without the presence of specific inhibitor to either PDGFR, Src-family kinases, PI3K or GPCR, we monitored the change in PDGF-stimulated ROS generation by capturing the fluorescence emitted from the live cells using confocal microscopy. DCF fluorescence intensity from the unstimulated cells was standardized to 100% and used as control. All other cells stimulated by PDGF with and without inhibitor were compared for fluorescent intensity relative to control cells. As shown in Figure 1, PDGF stimulation nearly doubled the fluorescent intensity over the control. However, inhibition of PDGFR (AG1296), Src-family kinases (PP1), or PI3K (LY294002) completely abolished (AG1296, 30 min), substantially attenuated (PP1 and LY294002, both 30 min), or partially attenuated (pertussis toxin, 1 h) fluorescence production.

### Effect of inhibition on PDGF, EGF and G protein coupled receptors on PDGF-stimulated signaling components in human lens epithelial (HLE) B3 cells

Western blot analyses were performed on lysates of cells treated with various concentrations of inhibitors to the PDGFR (AG1296), EGFR (AG1517) and GPCR (Ptx), either individually or in combination, in the presence of PDGF (1 ng/ml) for different periods of time (0, 10, 20, and 30 min). To ensure the concentrations of inhibitors and the incubation time used during the treatment were not harmful to the cells, LDH cytotoxicity assay was carried out in these cells and found no cell damage under the experimental conditions (data not shown).

### Effect of AG1296 (PDGFR inhibitor)

**Figure 2:**
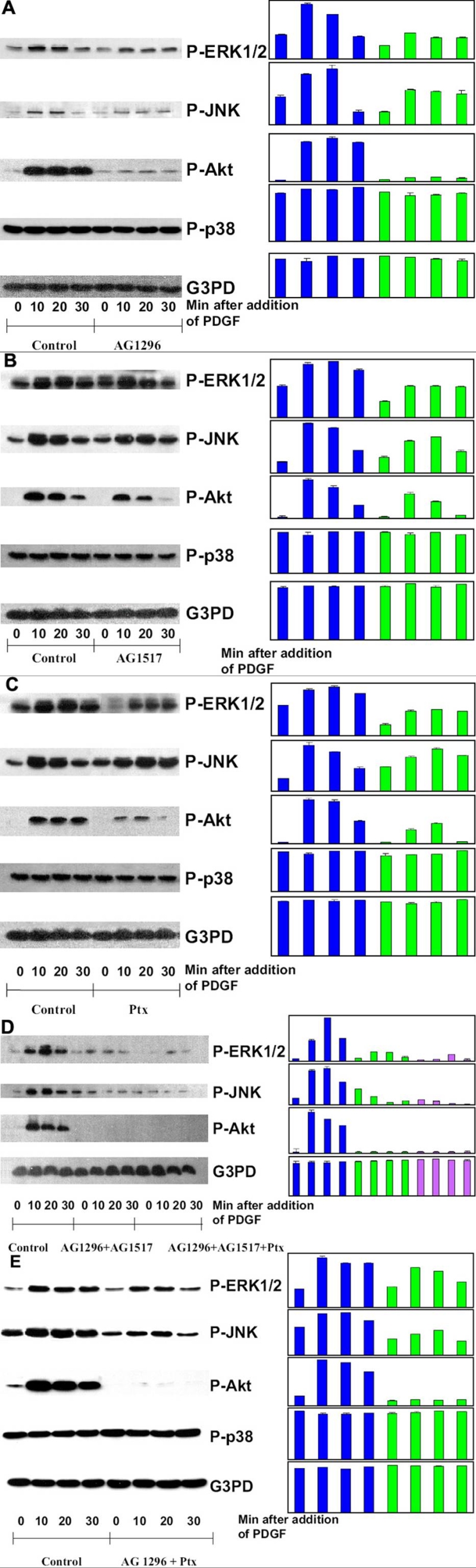
Effect of receptor inhibition on PDGF-activated MAP kinases in HLE B3 cells. Serum-deprived cells with and without inhibitor preloading were stimulated with PDGF (1 ng/ml) for 0, 10, 20, and 30 min, harvested and then lysed in lysis buffer. Cell lysates were applied on 10% SDS-PAGE, transblotted and probed with specific antibodies to P-ERK1/2, P-JNK, P-Akt, P-p38, and G3PD, respectively. **A**: Cells were stimulated with PDGF with and without preloading (2.5 h) of PDGF receptor inhibitor, AG1296 (20 μM). **B**: Cells were stimulated with PDGF with and without preloading (2.5 h) with EGF receptor inhibitor, AG1517 (1 μM). **C**: Cells were stimulated with PDGF with and without preloading (2.5 h) with pertussis toxin (Ptx, 500 ng/ml). **D**: Cells were stimulated with and without preloading (2.5 h) with AG1296 (20 μM) + AG1517 (1 μM), or AG1296 (20 μM) + AG1517 (1 μM) + Ptx (500 ng/ml). **E**: Cells were stimulated with and without preloading (overnight) with AG1296 (20 μM) + Ptx (250 ng/ml). Cells without treatment of inhibitors but with stimulation of PDGF (1 ng/ml) were used as the controls. The bar graph with averaged pixel values of the band intensities for each western blot is shown. Data presented are a typical representation of triplicate experiments.

We have demonstrated in the past that PDGF stimulation in HLE B3 cells activated ERK1/2 and JNK specifically [48]. The effect of these inhibitors on ERK1/2 and JNK were examined along with Akt, which is the downstream target for PI3K activation, and an important signaling component in cell survival in the lens [70]. As shown in Figure 2A, ERK1/2, JNK and Akt in the PDGF-stimulated control cells were all transiently activated, which began at 10 min and lasted until 30 min after PDGF stimulation. However, AG1296 (20 μM) severely suppressed Akt activation, substantially diminished the activations of ERK1/2 and JNK but did not affect P-p38 in cells stimulated with PDGF, indicating that AG1296 was specifically targeting ERK1/2 and JNK, but not the stress-associated p38. The constant level of G3PD in the immunoblot confirmed that equal amounts of proteins were loaded onto the gel.

### Effect of AG1517 (EGFR inhibitor)

An EGFR inhibitor was used to investigate if PDGF-stimulated cells may use the transactivation mechanism via EGF receptor. As shown in Figure 2B, inhibiting the cells with AG1517 (1 μM, 2.5 h) mildly diminishes PDGF-stimulated ERK1/2, JNK and Akt but has no effect on p38. These results suggest that HLE B3 cells have a transactivation system between PDGFR and EGFR, or that HLE B3 cells can partially rely upon the EGF receptor for PDGF signaling.

### Effect of pertussis toxin

Figure 2C depicts the western blot analysis of cells treated with pertussis toxin (500 ng/ml, 2.5 h), in which both ERK1/2 and JNK showed notably lower activation levels while Akt activation was extensively decreased. Again these inhibitions were very specifically targeted to the signal pathways of cell proliferation and cell survival, as there was no change on the level of phosphorylated p38. Therefore, the data suggest that crosstalk between the receptors of G-coupled protein and PDGF may exist, or the HLE B3 cells use GPCR in part for PDGF signaling.

### Effect of inhibition on multiple receptors

When the cells were inhibited simultaneously by PDGFR and EGFR inhibitors (AG1296 and AG1517), the downstream signals were either completely shutoff (Akt) or severely inhibited (ERK1/2 and JNK). Cells treated simultaneously with inhibitors to all three receptors (PDGF, EGF and GPC receptors) could no longer being stimulated by PDGF as the combined inhibition diminished the signals in ERK and JNK pathways to the basal levels and completely abolished Akt activation. These results are summarized in Figure 2D. Coinhibition of PDGFR and GPCR with 20 μM of AG 1296 and 250 ng/ml of Ptx in cells stimulated with PDGF also led to diminished activation in ERK1/2 and JNK, and severely suppressed P-Akt (Figure 2E).

### Inhibition effect of Src-family kinases and PI3K on the PDGF-stimulated signaling components in human lens epithelial (HLE) B3 cell

The target proteins immediately downstream of PDGF receptor activation were examined for their respective role in cell signaling. Src-family kinases were studied using a specific inhibitor, PP1, while PI3K was studied by using LY294002.

### Effect of Src inhibition by PP1

**Figure 3:**
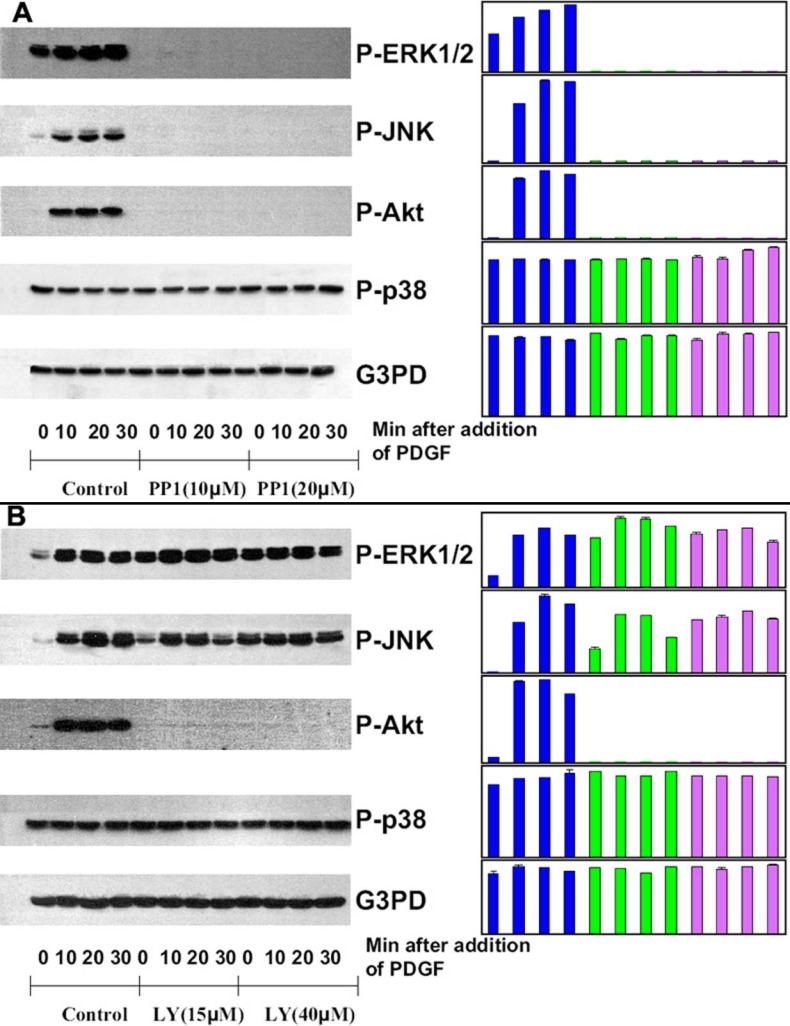
Effect of Src and PI3K inhibition on PDGF-stimulated MAP Kinases in HLEB3 cells. Serum-deprived cells with and without inhibitor preloading were stimulated with PDGF (1 ng/ml) for 0, 10, 20, and 30 min, harvested and then lysed in lysis buffer. Cell lysates were applied on 10% SDS-PAGE, transblotted and probed with specific antibodies to P-ERK1/2, P-JNK, P-Akt, P-p38, and G3PD, respectively. **A**: Cells were stimulated with PDGF with and without preloading (30 min) of Src inhibitor, PP1, at 10 μM or 20 μM. **B**: Cells were stimulated with PDGF with or without preloading (30 min) of PI3K inhibitor, LY294002 at 15 μM or 40 μM. Cells without treatment of inhibitor but with stimulation of PDGF (1 ng/ml) were used as the control. The bar graph with averaged pixel values of the band intensities for each western blot is shown. Data presented are a typical representation of 2-3 separate experiments.

Figure 3A depicts the western blotting of cells treated with PP1 (10 μM or 20 μM, 30 min), which clearly shows a near-complete suppression of ERK1/2, JNK and Akt activation in cells exposed to PP1 at both low and high concentrations. Similar to AG1296 above, PP1 inhibition is specific for ERK1/2 and JNK signaling components but not p38, which remained unchanged during PP1 inhibition.

### Effect of PI3K inhibition by LY294002

Figure 3B represents the western blotting for the lysates from cells treated with LY294002 (15 μM, or 40 μM, 30 min). As expected, Akt showed no activation signal in the presence of PI3K inhibitor, which appeared to be very specific to Akt and had little effect on other signaling pathways, including ERK1/2, JNK and p38.

### Effect of inhibition of PDGF receptors, Src-family kinases, PI3K and GPCR on cell proliferation in human lens epithelial (HLE) B3 cells

**Figure 4:**
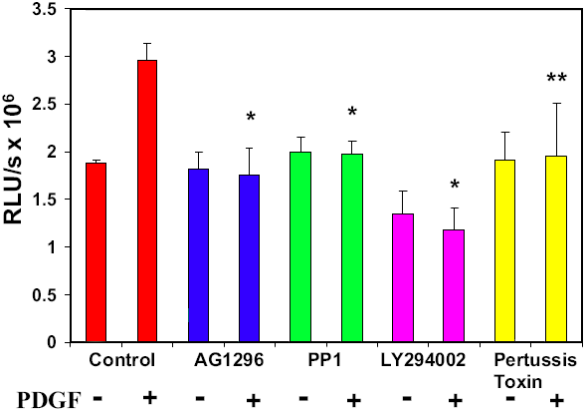
Determination of PDGF-stimulated cell proliferation in the presence or absence of inhibitors by BrdU incorporation assay. DNA synthesis induced by PDGF (1 ng/ml, 60 min) in cells with and without pretreatment of PDGF receptor inhibitor, AG1296 (20 μM, overnight), Src inhibitor, PP1 (10 μM, 30 min), PI3K inhibitor, LY294002 (30 μM, 30 min) or GPCR inhibitor, pertussis toxin (500 ng/ml, overnight) was measured by BrdU incorporation. Control cells (no inhibitor), were included for comparison. The data are expressed as relative luminescence unit (RLU)/sec, with mean±SD (n=12). The results are from 3 separate experiments. Each p value was obtained by using inhibitor (with PDGF) against control (with PDGF). The asterisk indicates a p<0.001 and the double asterisk denotes a p<0.026.

In our previous report [48], we have illustrated the importance of ROS generation towards cell proliferation. In this study, the inhibitory effect of PDGFR, Src-family kinase, PI3K and GPCR on cell growth was evaluated using BrdU incorporation assay. Figure 4 represents the quantity of incorporated BrdU in the cells treated with AG1296 (20 μM, 30 min), PP1 (10 μM, 30 min), LY294002 (30 μM, 30 min) or pertussis toxin (500 ng/ml, overnight) with or without PDGF (1 ng/ml) stimulation. It shows that PDGF-stimulated cell proliferation was nearly 30% higher than that of the unstimulated cells (control). Inhibiting PDGFR (AG1296), Src-family kinases (PP1), or GPCR (pertussis toxin) eradicated new DNA synthesis. However, inhibiting PI3K showed suppressed cell growth so much so that it was below the control. All these indicate that PDGFR and its receptor phosphorylated sites in binding with PI3K, Src-family kinases are essential for PDGF mitogenic action, and GPCR also plays an important role.

In another BrdU experiment, cell growth was monitored using individual specific inhibitors to PDGFR (AG1296, 20 μM for 2.5 h), EGFR (AG 1517, 1 μM for 2.5 h) or GPCR (Ptx, 500 ng/ml for 2.5 h), or in combined inhibitions of PDGFR + EGFR, or PDGFR + EGFR + GPCR. In comparison to the untreated control cells, each inhibitory condition showed a near total suppression of PDGF-stimulated cell growth (data not shown), consistent with the results on the attenuated MAPK signaling when the inhibitors were used under the same experimental conditions.

### The role of small GTP-binding proteins Rac and Ras in PDGF-stimulated ROS generation in human lens epithelial (HLE) B3 cells

**Figure 5:**
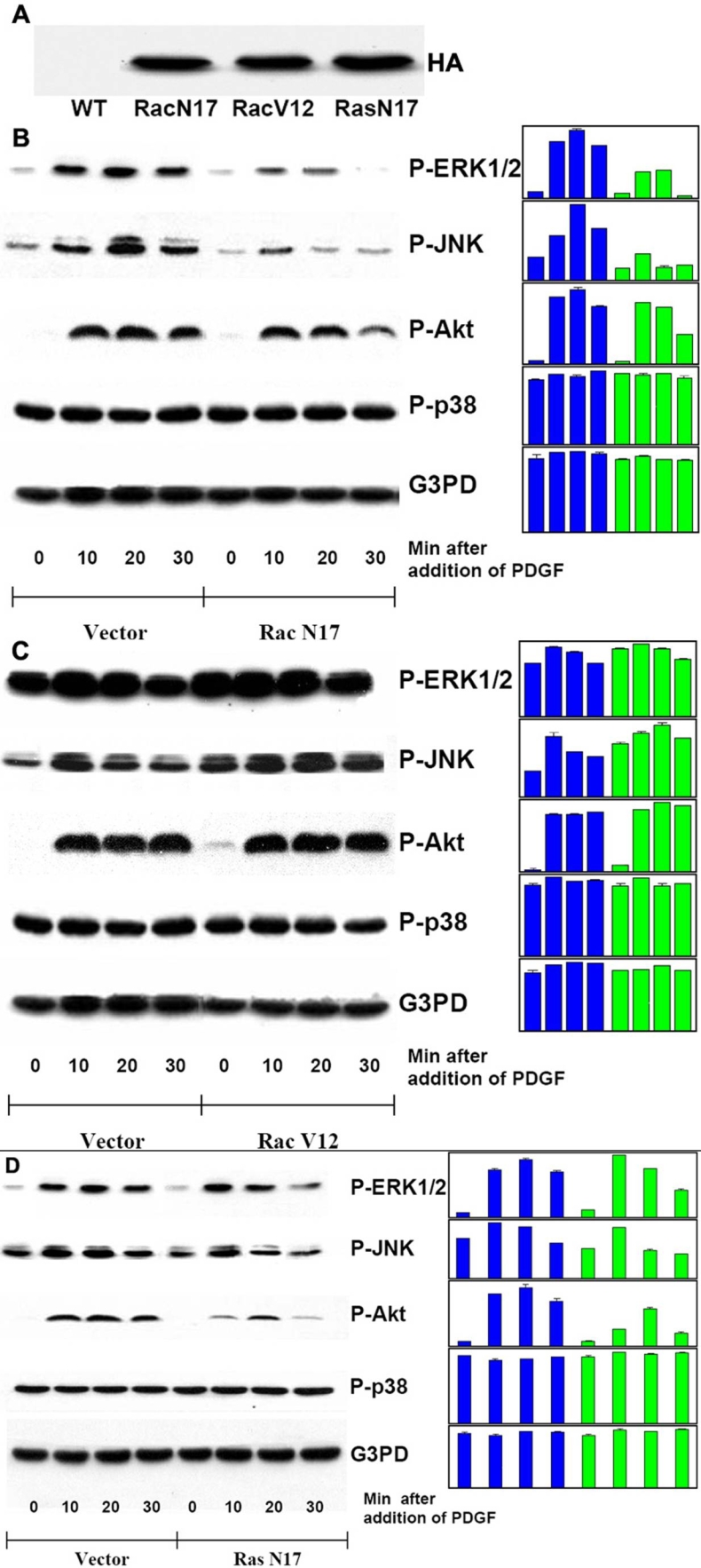
Western blot analysis of PDGF-activated MAP kinases in Ras or Rac transfected human lens epithelial B3 cells. Serum-deprived transiently transfected cells with and without inhibitor pretreatment were stimulated with PDGF (1 ng/ml) for 0, 10, 20, and 30 min, harvested and then lysed in lysis buffer. The vector-transfected cells were used as control. Cell lysates were applied on 10% SDS-PAGE, transblotted and probed with specific antibodies to HA, P-ERK1/2, P-JNK, P-Akt, P-p38, and G3PD. **A**: Wild type (WT) and transfected cells (Rac N17, Rac V12, Ras N17) were probed with anti-HA antibody. **B**: Vector- or Rac N17-transfected cells stimulated with PDGF were probed for P-ERK1/2, P-JNK, P-Akt, P-p38, and G3PD. **C**: Vector- or Rac V12-transfected cells stimulated with PDGF were probed for P-ERK1/2, P-JNK, P-Akt, P-p38 and G3PD. **D**: Vector- or Ras N17-transfected cells stimulated with PDGF were probed for P-ERK1/2, P-JNK, P-Akt, P-p38 and G3PD. The bar graph with averaged pixel values of the band intensities for each western blot is shown. Data presented are a typical representation of 2-3 separate experiments.

**Figure 6:**
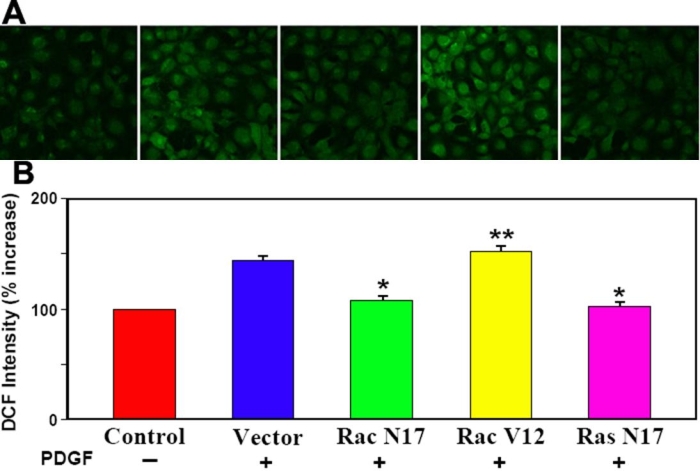
Confocal images of intracellular ROS generation upon PDGF stimulation in live Ras or Rac transfected cells. Serum starved transiently transfected cells were preloaded with DCFDA (50 μM) to capture the ROS generated upon PDGF (1 ng/ml) stimulation. Confocal cell images represent a random field after PDGF exposure. For details see Methods. **A**: Panels from left to right are: Negative control cells (vector transfected, no PDGF); positive control cells (vector transfected, with PDGF), dominant negative Rac transfected cells (Rac N17, with PDGF), constitutively active Rac tansfected cells (Rac V12, with PDGF), and dominant negative Ras transfected cells (Ras N17, with PDGF). **B**: Bar graph corresponding to the confocal images in **A**. The DCF fluorescent intensity was quantified using Confocal Assistant 4.02, and expressed as precent increase normalized to control (100%). The data are expressed as mean±SD with n=3. Each p value was obtained by using mutant transfectant against vector trasnfectant (with PDGF). The asterisk indicates a p<2x10^-5^ and the double asterisk denotes a p<0.03.

To examine the importance of Rac or Ras in PDGF-stimulated signaling, we used cells transfected with dominant negative Ras (Ras N17), dominant negative Rac (Rac N17) or constitutive active Rac (Rac V12) for evaluation. Western blot analysis was performed on HA-tag to ensure that HA-tagged cDNA was successfully transfected into HLE B3 cells. As shown in Figure 5A, all three transfectants (Rac N17, Rac V12, and Ras N17) contain HA-tag but no tag was found in the empty vector-transfected wild type (WT). The confocal images of vector, Rac N17, Rac V12 or Ras N17 transfected cells stimulated with PDGF (1 ng/ml) as compared with the un-stimulated control are shown in Figure 6A. The DCF fluorescence was clearly visible in vector-transfected cells stimulated with PDGF. In contrast, cells without PDGF stimulation at the same period of time (10 min) barely showed visible fluorescence. Upon PDGF stimulation, both the Rac N17- and Ras N17-transfected cells had only basal levels of fluorescence, while Rac V12-transfected cells were insensitive to PDGF, and had a consistently high level of fluorescence even in the absence of PDGF (Data not shown). The relative DCF intensity of each condition is summarized in Figure 6B.

### Small GTP-binding proteins Rac and Ras are important for MAPK activation stimulated by PDGF in human lens epithelial (HLE) B3 cells

Cell lysates from each transfectant, including Rac N17, Rac V12, Ras N17 and the control (vector only), were analyzed for the activations of ERK1/2, JNK, Akt, and p38. G3PD was also analyzed to ensure equal amounts of protein were applied. As shown in Figure 5B, Rac N17 transfected cells have shorter duration and weaker activations in ERK1/2, JNK, but no visible change in p38 when compared with the vector control. Rac N17 effect on Akt is minimal. Rac V12 transfected cells, on the other hand, showed prolonged activations and intensified signals for both ERK1/2 and JNK but had no effect on Akt or p38 (Figure 5C). Ras N17 transfectant showed similar effect on these signaling components as Rac N17, except that the non-functional Ras appeared to reduce activated Akt more effectively (Figure 5D). These results suggest that both Rac and Ras are essential regulators for PDGF signaling.

### The role of small GTP-binding proteins Rac and Ras on cell proliferation stimulated by PDGF in human lens epithelial (HLE) B3 cells

**Figure 7:**
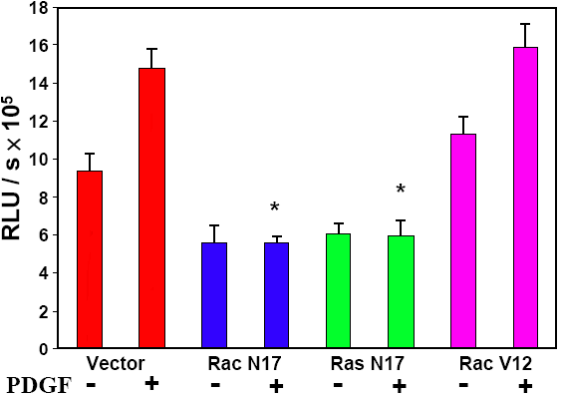
Determination of PDGF-stimulated cell proliferation in Ras or Rac transfected cells by BrdU incorporation assay. DNA synthesis was measured with or without addition of PDGF (1 ng/ml, 60 min) in transient tranfectants: vector (control), Rac N17, Ras N17, and Rac V12. The data are expressed as relative luminescence unit (RLU)/sec with mean±SD (n=12). The results are from 3 separate experiments. Each p value was obtained by using mutant transfectant (with PDGF) against vector transfectant (with PDGF). The asterisk indicates a p<1x10^-7^.

The effect of Rac and Ras on cell proliferation was demonstrated using BrdU incorporation assay. As shown in Figure 7, the control cells (vector only) increased DNA synthesis 30% after PDGF stimulation. Cells with dominant negative Rac (Rac N17) or Ras (Ras N17) had only 50% of the proliferation rate when compared with the control, and these transfectant cells are insensitive to PDGF stimulation. In contrast, the constitutively active Rac cells (Rac V12) exhibit higher growth rate than the control cells, with and without PDGF stimulation. Cell proliferation was also examined in these dominant negative and constitutively active cells using ^3^H-thymidine incorporation assay. The results showed suppression of DNA synthesis in Rac N17 or Ras N17 transfected cells with or without stimulation of PDGF, while no suppression was seen in Rac V12 transfected cells (data not shown). These findings suggest that both functional Rac and Ras are essential proteins for PDGF-stimulated cell proliferation.

## Discussion

PDGF ββ binding to the receptor in many cell types is known to induce several signaling pathways that lead to various cellular functions. It become clear in recent years, that PDGF mitogenic action is mediated by ROS generated during the process for the downstream signaling transduction cascades [35]. In our previous report [48], we demonstrated that the production of ROS in lens epithelial cells was triggered by PDGF, and linked with the subsequent transient ERK1/2 and JNK activations as well as cell growth. The current report not only confirmed our previous study but also further identified that PDGFR and its associated early binding proteins, including Src-family kinases, PI3K, and the small GTP-binding proteins of Rac and Ras, are essential elements for PDGF signaling induced cell proliferation. Our data suggest that the PDGF mitogenic action in human lens epithelial cells is regulated by the collective effort of these membrane-associated target proteins, similar to other cell types [55,71-75].

It is interesting that the inhibitor specific to PDGFR could eliminate PDGF-stimulated ROS generation, but could not inhibit ERK1/2 or JNK activation completely (Figure 2A). This phenomenon suggests that the cell may deliver the PDGF signal via other receptors [76]. It was shown in rat aortic vascular smooth muscle cells that PDGF ββ could transactivate EGF receptor to form a heterodimer between PDGFR and EGFR, which contributed to ERK1/2 activation. These authors also showed that antioxidants or Src inhibitor, but not PDGFR kinase specific inhibitor (AG 1295), could disrupt this receptor heterodimer, indicating that PDGFR kinase was not involved in the heterodimer activity. Furthermore, PDGF could also transactivate FGFR-1 and released bFGF in smooth muscle cells to enhance cell proliferation [77]. It is likely that a similar transactivation may occur in HLE cells, and the residual P-ERK1/2 signal during PDGFR inhibition observed in this study may be attributed to PDGF transactivated EGF or FGF signaling. However, HLE B3 cell is known to lack bFGF receptors and has a lower number of EGF receptors than PDGF receptors [78]. Thus, we speculate that the residual MAPK signaling could only be contributed from EGFR activation. Our current data did show that inhibition of EGFR negatively influenced the PDGF-stimulated ERK1/2, JNK and Akt signaling (Figure 2B) while inhibiting both receptors terminated Akt signal and rendered severely suppressed signals of ERK1/2 and JNK (Figure 2D). These data indicate that transactivation between PDGF and EGF receptors is likely functional in the lens epithelial cells.

It is intriguing that inhibition of GPCR by pertussis toxin, a G_i_α protein inhibitor, could partially suppress PDGF-induced ROS production, and the downstream ERK1/2, JNK, and Akt activations (Figure 1 and Figure 2C), albeit the inhibition was less effective as that of other inhibitors used in this study. This crosstalk between GPCR and PDGFββ-activated PDGFR is a novel finding in the lens epithelial cells. Recent studies have demonstrated a co-mitogenicity in a number of growth factors (for examples, EGF, IGF-1, and PDGF) to stimulate MAPK cascades via GPCR-mediated pathways in numerous cell types [58,79-83]. This new signaling system, called integrative signaling, is distinct from the transactivation of growth factors mentioned above, as it works via intimate binding of the components in two receptors between the receptor of protein tyrosine kinase (PTKR) and GPCR. Activation of this PTKR-GPCR complex is mediated by cytosolic Src and other adaptor proteins, to initiate MAPK signaling [79,84,85]. Disrupting GPCR would therefore destroy this complex and attenuate MAPK activation [45]. Indeed, we have observed a diminished MAPK signaling by co-inhibition of PDGFR and GPCR (Figure 2E), more so by co-inhibition of PDGFR, EGFR, and GPCR (Figure 2D), and a complete shutdown of MAPK signaling by inhibiting Src-family kinases (Figure 3A). All are indicative of the presence of this receptor-complex. If GPCR were associated with PDGFR binding through Src in our system, it would be reasonable to expect that inhibition of GPCR would affect the docking efficiency of Src and the downstream target proteins, including PI3K and MAPK pathways of ERK1/2 and JNK, as we have observed in this study.

Of all the inhibitors used, the PP1 specific inhibitor for Src-family kinases was the most effective as it could shutdown all three signaling pathways of ERK1/2, JNK, and Akt (Figure 3A). The inhibition was specific as p38 pathway was not affected. Src-family kinases are involved in many downstream signaling pathways [60] and they are also involved in the above-mentioned transactivation of heterodimer of PDGFR and EGFR [76]. Therefore, the strong inhibitory effect of Src-family kinases by PP1 in suppressing ROS production, MAPK activations and cell proliferation observed in our study is to be expected.

The importance of PI3K in PDGF mitogenic action has been established in various cell types. Bae et al. [15] has demonstrated that PDGF-induced ROS production requires PI3K activation, indicating that ROS generation is downstream from PI3K. It is known that PI3K activates Rac, which is an essential active component during NADPH oxidase activation [16]. Thus, both PI3K and Rac are necessary for ROS production. In our current study, the specific inhibitor to PI3K (LY294002) effectively eliminated the fluorescent production from intracellular ROS in HLE cells during PDGF stimulation (Figure 1). Inhibiting PI3K also eliminated Akt activation as expected, but it had no effect on ERK1/2 and only weakly attenuated P-JNK (Figure 3B). It is known that activation of JNK is downstream from Rac [73], thus, it is reasonable to expect that some inhibitory effect on JNK activation would occur. However, inhibition of PI3K may have limited effect on P-ERK1/2 as activation of ERK1/2 can be initiated from several sources besides PI3K, including Ras, and to some degree from βγ heterodimer-activated receptor tyrosine kinases during GPCR activation [79,80].

Cells transfected with constitutively active Rac plasmid (Rac V12), showed a similar increase in fluorescence as the vector transfected cells (44-50%) during PDGF stimulation, but the basal level of fluorescence was considerably higher in the Rac V12 than that of vector transfected cells (data not shown). In contrast, cells transfected with dominant negative Rac (Rac N17) could not produce any ROS upon PDGF stimulation (Figure 6). As expected, genetic manipulation of Rac changed the up and down signals of P-JNK but had less effect on ERK1/2 (Figure 5B). Dominant negative Ras also prevented ROS generation and shortened the duration of activated MAPK pathways, except p38, indicating that Ras occupies the initial stage of the PDGF signaling system.

**Figure 8:**
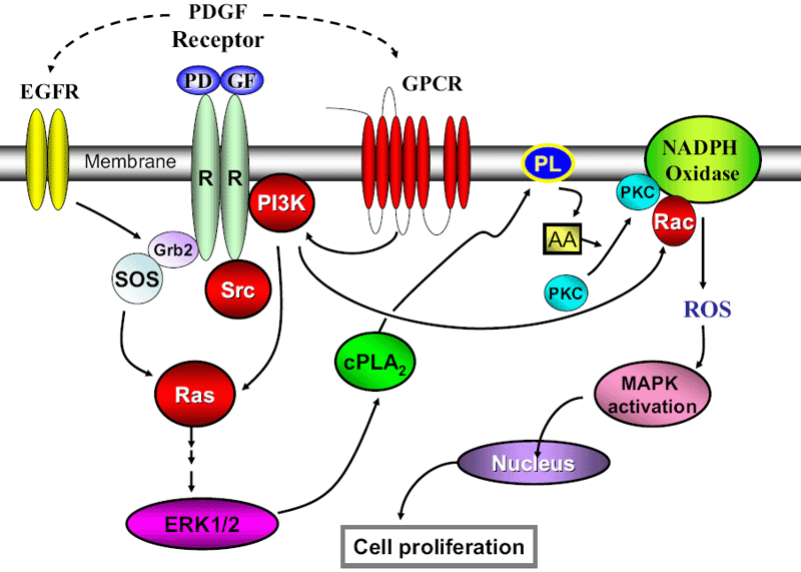
The proposed mechanism of PDGF signaling in the lens epithelial cells. The solid line indicates the known or published pathway and the dashed line represents proposed new pathway. In the figure, EGF denotes Epidermal growth factor, GPCR indicates G protein coupled receptor, PI3K indicates phosphatidyl inositol-3-kinase, cPLA_2_ denotes cytosolic phospholipase 2, PL indicates Phospholipid, AA denotes Arachidonic acid, PKC indicates protein kinase C, and ROS denotes Reactive oxygen species.

Taken together, our data indicate that ROS generation, the downstream MAPK activation, and the cell proliferation upon PDGF stimulation require the concerted effort of the upstream membrane-associated components of PDGF receptor kinase, Src-family kinases, PI3K and the GTP-binding proteins of Rac and Ras. We speculate that GPCR and EGFR may also play a regulatory role in this process. These findings provide an insight into the mechanistic regulatory system for the complex PDGF signaling pathway in the lens epithelial cells. Our previous report [51], which focused on the pathways downstream from the factors covered in this study, demonstrated that PDGF-stimulated ERK1/2 activation induced arachidonic acid release to facilitate ROS production from NADPH oxidase. We have thus proposed that the mechanism of PDGF stimulation may use a positive feedback loop of PDGFR-ERK1/2-arachidonic acid-ROS-ERK1/2-cell proliferation. Together with our findings on the regulatory components upstream from our previous work, we propose the following mechanism of PDGF signaling in the lens epithelial cells as depicted in Figure 8.
